# Intravenous Thrombolysis for Acute Ischemic Stroke Following Cervical Epidural Injection: A Case Report

**DOI:** 10.7759/cureus.81518

**Published:** 2025-03-31

**Authors:** Cattien N Phan, Muhammad Tar, Theresa LaBarte

**Affiliations:** 1 Department of Neurology, Loma Linda University Medical Center, Loma Linda, USA; 2 Department of Neurology, Riverside University Health System Medical Center, Moreno Valley, USA

**Keywords:** acute ischemic stroke (ais), epidural injection, intravenous thrombolysis, neuraxial procedure, stroke

## Abstract

A recent lumbar puncture (LP) is a relative contraindication for intravenous thrombolysis (IVT) due to the potential for the development of spinal hematoma. While there have been case reports of IVT administered without complications following LP or epidural anesthesia, there is limited data overall regarding the safety of IVT for acute ischemic stroke (AIS) in patients who have undergone a neuraxial procedure. We present a case report of an 83-year-old man who presented with a sudden onset of symptoms consistent with left middle cerebral artery (MCA) stroke after receiving a cervical epidural injection (CEI) earlier that same day. The patient received IVT after a comprehensive examination, including brain imaging that showed no intracranial hemorrhage and confirmed a large vessel occlusion (LVO), and a thorough assessment of the risks and benefits. There were no reported complications post treatment, and the patient showed improvement in symptoms on follow-up visits. This case report highlights the safe and successful administration of IVT for AIS following a recent CEI.

## Introduction

The use and efficacy of intravenous thrombolysis (IVT) in the management of acute ischemic stroke (AIS) are well established. Following the landmark National Institute of Neurological Disorders and Stroke (NINDS) trial, which demonstrated improved long-term outcomes in patients treated with recombinant tissue plasminogen activator (tPA) for AIS, the US Food and Drug Administration (FDA) approved tPA for AIS in 1996 [1,2]. The inclusion and exclusion criteria for IVT have evolved over time. However, most guidelines, including the FDA label for IV tPA, do not explicitly address the use or safety in the context of recent neuraxial procedures, such as lumbar puncture (LP) or spinal epidural injection [3]. According to a published statement by the American Heart Association (AHA) and American Stroke Association (ASA), IVT may still be considered for patients with AIS, even if they have undergone a lumbar dural puncture within the preceding seven days [4]. Thus, LPs and similar procedures are not absolute contraindications to IVT. Clinicians must carefully evaluate the potential risks and benefits of IVT in these scenarios to guide effective clinical decision-making. This article was previously presented as a meeting abstract at the 2024 Society of Vascular and Interventional Neurology Annual Meeting on November 21, 2024.

## Case presentation

An 83-year-old right-handed man with a medical history of dyslipidemia, prior stroke with residual right arm weakness and numbness, and chronic right arm paresthesia secondary to cervical spondylosis presented with a sudden onset of right facial droop and aphasia. Despite his comorbidities, the patient was ambulatory and independent in activities of daily living (ADLs) prior to presentation, with a modified Rankin Scale (mRS) score of 1. He was on daily aspirin and clopidogrel for secondary stroke prevention.

On arrival at the emergency department (ED), a stroke code was activated due to acute focal neurological deficits. Neurological examination revealed worsened right upper extremity weakness compared to baseline, along with new-onset right facial droop, dysarthria, and expressive aphasia. The patient’s National Institutes of Health Stroke Scale (NIHSS) score at presentation was 12 (Table 1). Clinical symptoms and examination findings were strongly indicative of an AIS localized to the left middle cerebral artery (MCA) territory.

**Table 1:** Score breakdown of NIHSS on the day of symptom onset and at the three-month video follow-up. NIHSS: National Institutes of Health Stroke Scale

Items	Day of Symptom Onset	3-Month Follow-Up
Level of consciousness	0: alert and keenly responsive	0: alert and keenly responsive
Answering questions	2: answers neither questions correctly	0: answers both questions correctly
Following commands	0: performs both tasks correctly	0: performs both tasks correctly
Best gaze	0: normal	0: normal
Visual fields	0: no visual loss	0: no visual loss
Facial palsy	2: partial paralysis (total or near-total paralysis of the lower face)	2: partial paralysis (total or near-total paralysis of the lower face)
Left motor arm	0: no drift and limb holds for full 10 seconds	0: no drift and limb holds for full 10 seconds
Right motor arm	1: drift, limb holds but drifts down before full 10 seconds, and does not hit bed	1: drift, limb holds but drifts down before full 10 seconds, and does not hit bed
Left motor leg	0: no drift and limb holds for full 10 seconds	0: no drift and limb holds for full 10 seconds
Right motor leg	1: drift, limb holds but drifts down before full 10 seconds, and does not hit bed	0: no drift and limb holds for full 10 seconds
Limb ataxia	0: absent	0: absent
Sensory	1: mild-moderate sensory loss and can sense being touched	1: mild-moderate sensory loss and can sense being touched
Best language	3: mute, global aphasia, and no usable speech or auditory comprehension	1: mild-moderate aphasia and some obvious changes
Dysarthria	2: severe dysarthria and unintelligible slurring or out of proportion to dysphasia	1: mild-moderate dysarthria and slurring but can be understood
Extinction or inattention	0: no abnormality	0: no abnormality
Total score	12	6

**Figure 1:**
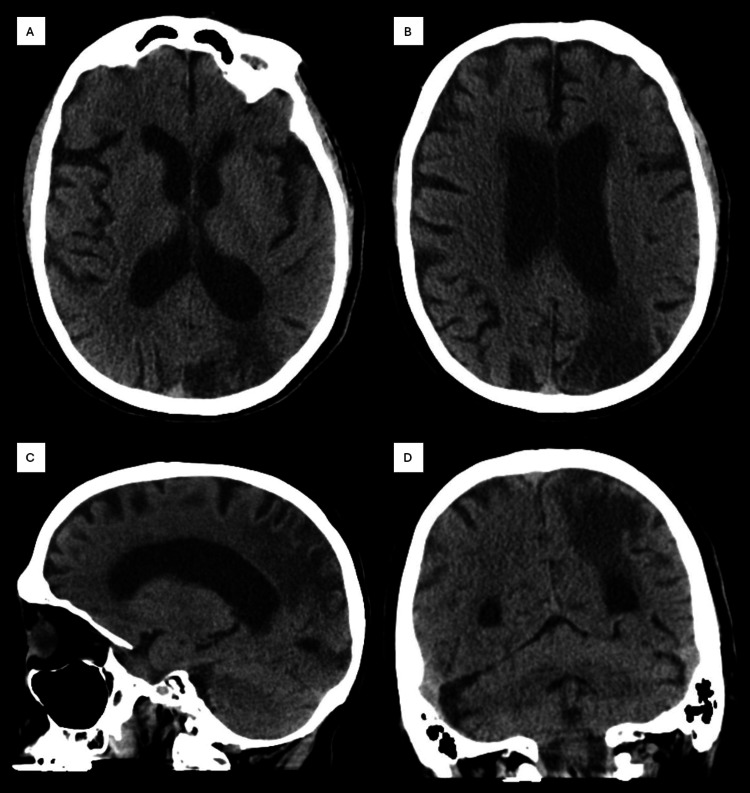
Non-contrast CT of the head obtained in the ED. Non-contrast CT scan of the head in the axial (A and B), sagittal (C), and coronal (D) views. Findings showed no evidence of intracranial hemorrhage and a remote infarct in the left posterior parieto-occipital region. CT, computed tomography; ED, emergency department

**Figure 2:**
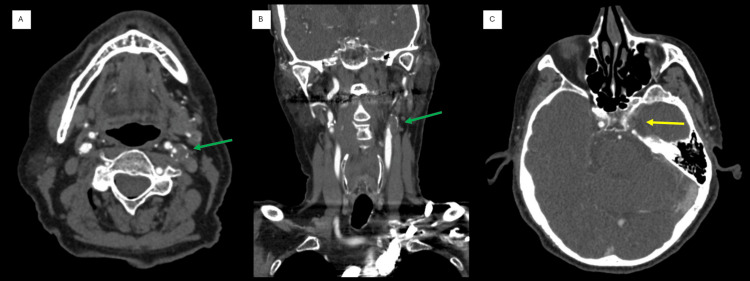
CT angiogram of the head and neck. CT angiogram of the neck showing the left internal carotid artery (ICA) occlusion just at the origin (green arrows) in the axial (A) and coronal (B) views. Axial CT angiogram of the head (C) showing the occlusion extending to the cavernous section of the left ICA (yellow arrow). CT: computed tomography

In the ED, vital signs included a blood pressure of 152/96 mmHg and a point-of-care glucose level of 115 mg/dL. Laboratory results, including a comprehensive metabolic panel, complete blood count, prothrombin time, and international normalized ratio (INR), were all within normal limits. Non-contrast computed tomography (CT) of the head revealed no evidence of intracranial hemorrhage but showed an old infarct in the left posterior parieto-occipital region, which was consistent with known clinical history (Figure 1). A CT angiogram of the head and neck demonstrated the occlusion of the left internal carotid artery (ICA) starting at the origin and extending to the cavernous segment (Figure 2).

The patient presented approximately 2.5 hours from the last known well time, which fell within the time window for IVT. Earlier that day, the patient had undergone a cervical epidural injection (CEI) for chronic right arm paresthesia. Notably, he had temporarily discontinued aspirin and clopidogrel five days prior to the procedure. The medical team contacted the patient’s son, a physician assistant, who was present during the CEI and therefore was able to provide additional details about the procedure. The CEI was performed at the C7 level and was confirmed to be uncomplicated, with clinical observations indicating that the dura had not been punctured. Aside from the recent CEI, the patient had no other absolute or relative contraindications to IVT.

Neurointerventional radiology (NIR) was consulted to evaluate the safety of IVT in the context of the recent CEI. After an extensive discussion about the indications, risks, and benefits of intravenous (IV) thrombolysis, informed consent was obtained from the patient’s family, including a pointed discussion regarding the theoretically increased risk of harm as compared to typically quoted IVT risks in the context of the patient’s recent procedure. Intravenous tenecteplase (TNK) 16.5 mg was administered according to our institutional IVT protocol. The patient was transferred to a thrombectomy-capable stroke center. At the stroke center, he underwent balloon angioplasty and stenting of the left internal carotid artery (ICA).

Post-procedural magnetic resonance imaging (MRI) of the brain confirmed an acute ischemic infarct in the left frontal lobe. The patient remained stable without complications from TNK administration. He was discharged home on optimized medical management, including dual antiplatelet therapy, and was referred to home health physical therapy.

At a one-month follow-up telephone call, the patient’s family reported improvement in his strength with physical therapy. However, he had residual aphasia that made communication challenging. By the three-month follow-up video visit, the patient had regained independence with most activities of daily living (ADLs). Because he does require assistance with some ADLs, such as preparing meals, the mRS is 1. The physical examination is limited due to the nature of the video visit. The NIHSS obtained was 6 (Table 1). While his speech had improved, he continued to experience some difficulty with naming and multistep tasks.

## Discussion

For our patient, several factors influenced the decision to proceed with IVT. Firstly, the patient’s history and clinical presentation strongly suggested an acute left MCA stroke. One of the presenting symptoms, aphasia, is a cortical sign that indicates a high likelihood of large vessel occlusion (LVO) and is recognized as a disabling stroke symptom [5]. Imaging with a CT angiogram of the head and neck confirmed the LVO (Figure 2). Secondly, a discussion with a family member with a medical background who relayed direct clinical observations during the CEI indicated that the dura was not punctured. This information was critical in assessing the risk of bleeding with IVT and suggested a lower likelihood of spinal hematoma development. Additionally, the case was extensively discussed with the neurointerventional radiology (NIR) team, including potential interventions and management strategies for complications. Finally, these considerations were thoroughly explained to the patient’s family, who provided informed consent for IVT. Additionally, thrombectomy is not available at our hospital. The time required to transfer the patient to a thrombectomy-capable center could place the patient outside the recommended time window for IVT.

Our case report has the following limitations. Medical records from the hospital that the patient was transferred to were not available for our review. It would be helpful to better understand the case if we had further information regarding the patient’s examination 24-36 hours post IVT and any imaging obtained post IVT to evaluate for spinal hematoma or post procedure to evaluate stent placement. Due to transportation problems, the patient was not able to follow up in person in the clinic. The assessment of the patient’s functional status was based mainly on subjective history at one month and a visual examination through a video call at three months.

To better understand the safety and risks of IVT in AIS cases involving recent neuraxial procedures, we conducted a mini literature review using PubMed with the search terms “acute ischemic stroke”, “IV thrombolysis”, “epidural injection”, “lumbar puncture”, and “spinal hematoma” for articles from 2007 to December 1, 2024. The inclusion criteria included incidences of spinal hematomas with IVT for the treatment of AIS in the setting of a neuraxial procedure. Book chapters, letters, and editorials were excluded.

The review identified four case reports of spinal hematoma after tPA administration and one case of cervical epidural hematoma following TNK administration in AIS patients (Table 2). These reports highlight the risk of spinal hematoma with IVT, even in the absence of recent neuraxial procedures [6-10]. There were only two case reports documenting uncomplicated and successful IVT administration for AIS in patients with recent LP or epidural catheter placement (Table 2). Baciewicz et al. described a 66-year-old man who developed ischemic stroke after epidural catheter placement for abdominal surgery and received IV tPA one hour after catheter removal, with favorable recovery [11]. Similarly, Shekhar et al. reported a 59-year-old woman who developed acute focal neurological symptoms during an LP and was treated with IVT without complications [12].

**Table 2:** Results of the mini literature review. Characteristics of articles identified from the focused mini literature review. IVT, intravenous thrombolysis; tPA, tissue plasminogen activator; TNK, tenecteplase; HTN, hypertension; DM, diabetes mellitus; HLD, hyperlipidemia; BLE, bilateral lower extremity; NIHSS, National Institutes of Health Stroke Scale; IV, intravenous

Article	Type of Study	Presentation	Type of IVT	Complications
Kim et al., 2008 [6]	Case report	A 49-year-old woman with paroxysmal atrial fibrillation presented with acute left hemiparesis and slurred speech. NIHSS was 8	tPA. Note: IV heparin was started for atrial fibrillation 24 hours post tPA	Anterior spinal subdural hematoma from C7 to T2 confirmed on imaging >24 hours after tPA
Yeo et al., 2009 [7]	Case report	A 62-year-old woman with no known cardiovascular risk factors presented with acute right-sided weakness. NIHSS was 8	tPA	Cervical epidural hematoma at C4-C7 confirmed on imaging the next day
Liebkind et al., 2010 [8]	Case report	An 80-year-old man with HTN and DM who had a sudden fall at home was found to have acute right hemiparesis. NIHSS was 5	tPA	Spinal epidural hematoma from C6 to T6 approximately two hours after tPA
Ghadirpour et al., 2014 [9]	Case report	A 75-year-old man with uncontrolled HTN and DM with acute right hemiparesis. NIHSS was 8	tPA	Cervical epidural hematoma on the third day after tPA
Zhao et al., 2024 [10]	Case report	A 71-year-old man with HTN presented with acute left-sided weakness and left facial droop	TNK	Cervical spinal subdural hematoma approximately two hour and 40 minutes after IVT
Baciewicz et al., 2017 [11]	Case report	A 66-year-old man with HTN, DM, HLD, traumatic amputation of left lower leg, and anal melanoma received an epidural catheter. Two days later, he developed acute aphasia, right-sided weakness, and neglect. NIHSS was 9	Epidural catheter was removed; tPA was administered one hour later	No complications
Shekhar et al., 2018 [12]	Case report and conference abstract	A 59-year-old woman with HTN, migraine, and chronic BLE weakness developed acute left-sided weakness and numbness during lumbar puncture. NIHSS was 3	tPA	No complications. The patient did not develop back pain or signs and symptoms to suggest epidural hematoma

However, no case reports or observational studies were identified that specifically address the risk of spinal hematoma with IVT in patients who recently underwent lumbar dural puncture or similar procedures. Given the limited available data, further studies are warranted to establish the incidence of spinal hematoma and the safety profile of IVT in AIS patients following neuraxial procedures.

## Conclusions

According to the AHA/ASA guidelines, lumbar dural puncture within the preceding seven days is a relative contraindication to IVT for AIS. Clinicians must carefully weigh the benefits and risks of IVT in such cases. Our case report of the safe and successful administration of IVT for AIS following a recent CEI with resulting favorable recovery and no discernible treatment-related adverse events highlights the complexity of the clinical decision-making for these cases. The decision for IVT for our patient was made following the confirmation that the dura was not punctured and with the consideration that symptoms were disabling. Further studies are necessary to establish the safety of IVT after neuraxial procedures and provide guidance for clinical management.
